# What remains from living cells in bacterial lysate-based cell-free systems

**DOI:** 10.1016/j.csbj.2023.05.025

**Published:** 2023-05-24

**Authors:** Léa Wagner, Matthieu Jules, Olivier Borkowski

**Affiliations:** Université Paris-Saclay, INRAE, AgroParisTech, Micalis Institute, 78350, Jouy-en-Josas, France

**Keywords:** Cell-free, *E. coli*, Prototyping, Adaptation, Homeostasis, Spatial organization, Microfluidics

## Abstract

Because they mimic cells while offering an accessible and controllable environment, lysate-based cell-free systems (CFS) have emerged as valuable biotechnology tools for synthetic biology. Historically used to uncover fundamental mechanisms of life, CFS are nowadays used for a multitude of purposes, including protein production and prototyping of synthetic circuits. Despite the conservation of fundamental functions in CFS like transcription and translation, RNAs and certain membrane-embedded or membrane-bound proteins of the host cell are lost when preparing the lysate. As a result, CFS largely lack some essential properties of living cells, such as the ability to adapt to changing conditions, to maintain homeostasis and spatial organization. Regardless of the application, shedding light on the black-box of the bacterial lysate is necessary to fully exploit the potential of CFS. Most measurements of the activity of synthetic circuits in CFS and *in vivo* show significant correlations because these only require processes that are preserved in CFS, like transcription and translation. However, prototyping circuits of higher complexity that require functions that are lost in CFS (cell adaptation, homeostasis, spatial organization) will not show such a good correlation with *in vivo* conditions. Both for prototyping circuits of higher complexity and for building artificial cells, the cell-free community has developed devices to reconstruct cellular functions. This mini-review compares bacterial CFS to living cells, focusing on functional and cellular process differences and the latest developments in restoring lost functions through complementation of the lysate or device engineering.

Lysate-based cell-free systems (CFS) prepared from bacteria are commonly used as testbeds to *in vitro* characterize synthetic circuits before *in vivo* implementation. The goal of lysate preparation is to extract a bacterial cytoplasm that is as well preserved as possible so that most cellular compounds, like proteins and metabolites, remain effective. As a result, the transcription and translation processes needed to produce proteins from DNA are efficient in CFS, despite the damages caused by the physical or chemical disruption of the cell [1], [2], [3]. However, to produce a large amount of protein, the lysate still requires additional compounds such as a precursor for ATP regeneration, a crowding agent, salts, and building blocks like NTPs or amino acids [4], [5], [6]. Other essential cellular processes taking place within living cells like glycolysis are preserved as well, while many may be lost [7], [8]. For instance, proteins that are embedded in or bound to the membrane are discarded, which calls into question the maintenance of respiratory functions in CFS. Beyond the loss of particular proteins, removing the membrane also leads to the loss of the cell's ability to adapt to changing conditions and to maintain homeostasis in non-equilibrium thermodynamics by exchanging molecules with the outer medium. That said, the interest in CFS as testbed platforms from the 1960 s to the present day is due to the ease of expressing proteins in a fully open, controllable environment [9].

The first use of lysates was reported in 1897 by Eduard Buchner who discovered that cell extract from macerated yeast can ferment sugar into alcohol and carbon dioxide. This discovery led to the drop of the protoplast theory in favour of the enzyme theory and the birth of biochemistry [10]. Cell-free systems were first used in the context of molecular biology in the 1960′s by Nirenberg and Matthaei to decode the 64 different codons in the genetic code [11], [12]. In these pioneering studies, the cells were broken by griding with alumina and the resulting lysate was supplemented with a mixture of amino acids, ATP, GTP, CTP, UTP, PEP and PEP kinase. Despite the fact that the RNA synthesis process was not yet understood and the translation process was still being investigated, this was a ground-breaking experiment that demonstrated the potential for using cell lysate to control and replicate fundamental processes that occur in living cells.

In the 2000s and 2010s, CFS' potential to mimic the functions of living cells was exploited in synthetic biology for prototyping genetic circuits and metabolic pathways. Different studies reported significant correlations between *in vitro* and *in vivo* promoter and ribosome binding site (RBS) strengths [13]. This allowed optimizing the expression of complete metabolic pathways [14], [15] and led to the development of genetic tools to predict the cost of protein production within living cells [16].

Whether for elucidating molecular biological mechanisms or for harnessing the potential of cell-free for prototyping, it is necessary to identify the similarities and differences between CFS and living cells. Recent efforts by the synthetic biology community have been aimed at independently reconstructing the different cellular functions lost during the preparation of CFS. Indeed, one of the synthetic biology community ambitions is to build a complete synthetic cell. This pursuit of reconstructed cells has led to the examination of the remaining, lost, and potentially rebuildable functions in CFS. The objective of this mini-review is to compare *Escherichia coli* CFS to living cells in terms of functions and cellular processes, and to give an overview of (i) the protein production processes and metabolism that mainly remain functional in CFS and (ii) the functions of living cells that can be restored by complementation of the lysate or by device engineering.

## 1 *In vivo* functions that are mostly preserved in lysate-based cell-free systems

### 1.1 DNA replication

**Fig. 1:**
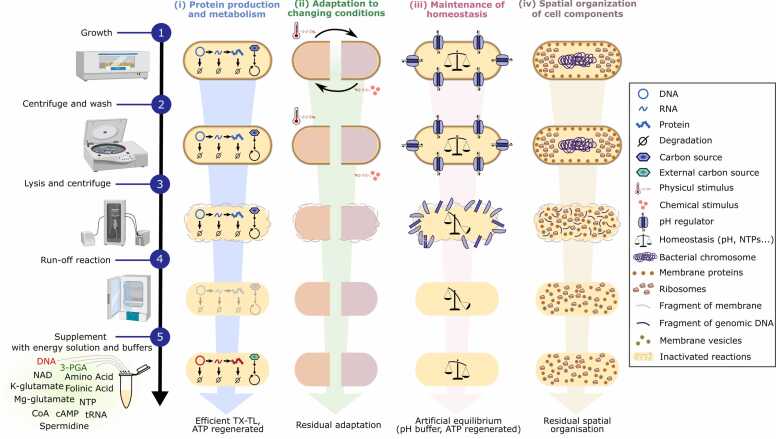
**Characterization of the *in vivo* features remaining in Bacterial lysate-based cell-free system**s **(CFS) across the main steps of its preparation.** The 5-step protocol on the left is pictured with the equipment needed to produce CFS. (i) Regarding protein production and metabolism, CFS is a well-recognised platform for gene expression in which some metabolic pathways like those regenerating ATP are still functional. (ii) All the dynamics systems originally present in a living cell (response to an external stimulus, sensor and transporter ensuring exchanges with the external medium) does not exist anymore in CFS. (iii) While the living cells maintain homeostasis in a non-equilibrium steady state, CFS relaxes to biochemical equilibrium. Only some parameters such as pH and NTPs concentrations are stabilised for a few hours and minutes respectively. (iv) As it is a dilute and well-mixed reaction environment, CFS no longer presents any spatial organisation. The machine icons come from BioRender.com. TX-TL: Transcription and translation.

During the preparation of the lysate, physical or chemical disruption of the cells is commonly performed (Fig. 1, step 3 on the left). This step is known to cause damage to the genome, resulting in loss of integrity and the formation of multiple fragments [17]. During the centrifugation process, which is used to remove most of the debris, there is no evidence of loss of the genome fragments. However, when comparing the yield of ^14^C-leucine incorporation into synthetized protein in crude extract before or after centrifugation without a DNA template, the first centrifugation step has been shown to reduce background expression (*i.e*., protein production from remaining genome fragments) as compared to no centrifugation [18]. The run-off step (Fig. 1, step 4 on the left), which involves a 1–1.5 h incubation at 37 °C of the lysate, provides conditions for the possible digestion of the DNA fragments [19], [20]. It is well established in the literature that linear DNA fragments are rapidly digested in cell-free reactions when the lysate is made from standard *E. coli* strains [20]. As a result, several studies have focused on streamlining *E. coli* strains to optimize protein production from PCR-amplified genetic circuits, in particular by knocking out the *recBCD* genes to prevent DNA degradation [20], [21], [22]. It is an open question whether the use of these strains results in the presence of remaining genomic DNA fragments in CFS despite the run-off step and whether these fragments affect the transcription-translation process. Besides, cells cultured at low temperature (20–34 °C) or adding inhibitors such as GamS, Chi DNA, and certain small molecules also help decrease DNA degradation [20], [23].

**Fig. 2:**
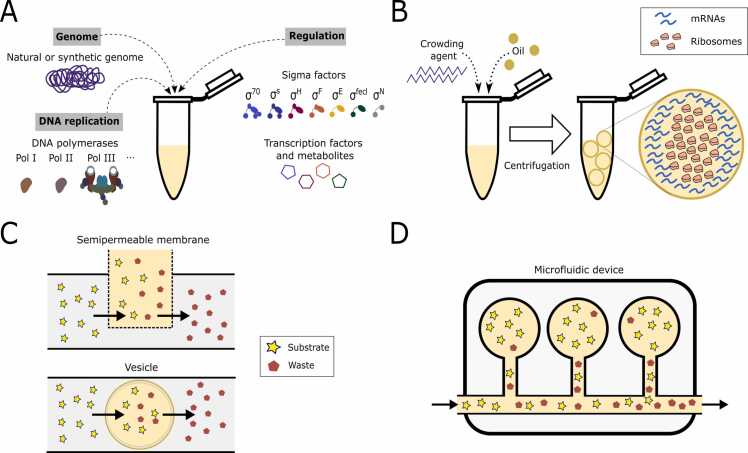
**Devices developed to restore structures and functions that are defective in CFS.** (A) Almost all lost functions related to the central dogma can be restored by adding missing purified molecules. (B) Crowding agents coupled with encapsulation into lipidic vesicles can be used to partially restore spatial organization. (C) In an attempt to restore homeostasis and adaptive behaviors, porous membranes or phospholipid vesicles can be used to supply the CFS reaction with substrate while rejecting waste to prevent deleterious accumulation. (D) An alternative way to achieve this goal is to use microfluidic devices.

Although the *E. coli* genome DNA is lost when using standard strains, replication of DNA material appears to be measurable in cell-free systems. Early experiments in the 1970s and 1980s demonstrated that plasmid replication occurs in cell-free systems [24], [25], [26]. Plasmids with ColE1 or R6K replication origins showed initiation, elongation, and termination of replication and DNA synthesis was monitored over time [27], [28]. Although, the R6K plasmids used at the time to study replication contain some genes required for replication initiation, such as *pir* (the replication initiator for R6K), host proteins such as DnaA, IHF, Fis and the DNA polymerase complex are essential for the replication initiation and elongation steps. The R6K replication efficiency is low in CFS, suggesting that the replication process may not be stable or that DNA is degraded as it is amplified [28]. Moreover, as dNTPs are not added to final cell-free reactions in the current protocols, we can state that replication remains inactive in CFS as recently suggested [9]**.** It is worth noting that bottom-up reconstruction of DNA replication has been achieved in cell-free systems (Fig. 2A) [29], [30], [31], [32]. This opens the way for the prototyping of self-replicating genetic sequences and potentially for new applications [3], [33].

### 1.2 Transcription and transcriptional regulation

The core RNA polymerase (RNAP) remains present in the lysate and active in the final cell-free reaction. Its presence has been confirmed through the use of native promoters that control gene encoding GFP, coupled with an aptamer that specifically binds to a dye to provide a fluorescent signal linked to mRNA concentration [34], [35]. Although the absence of the endogenous genome in CFS prevents RNAP regeneration, the stability of the bacterial RNAP complex is sufficient to produce mRNA even ten hours after the reaction begins [35], [36]. However, the bacterial RNAP remains less efficient than the T7 bacteriophage RNAP, which is commonly supplied to CFS using a T7 RNAP-encoding *E. coli* strain, like BL21 DE3, to make the lysate [34], [37].

An essential question that arises when studying transcription in CFS is to what extent the regulations naturally occurring *in vivo* are maintained. In bacteria, the regulation of transcription is an essential mechanism of adaptation to the environment, ensured both by sigma factors (σ) and transcription regulators. In *E. coli*, seven sigma factors naturally control gene expression across environmental growth conditions: σ^70^/σ^D^ for housekeeping genes during exponential growth, σ^54^/σ^N^ for growth in limiting nitrogen condition, σ^38^/σ^S^ for survival during stationary phase, σ^32^/σ^H^ for the heat shock response, σ^28^/σ^F^ for chemotaxis, σ^24^/σ^E^ for refolding denatured proteins, and finally σ^FecI^ for iron citrate transport [38]. The benefit of using the native RNAP in CFS is that it naturally interacts with sigma factors and regulators. Indeed, the sigma factors are present in the cell-free reaction and their presence depends on the conditions used to prepare the lysate [39], [40]. This point has been studied in the different versions of the *E. coli* TXTL toolbox developed by Noireaux’s lab [34], [41], [42], [43]. Promoters specific to each sigma factor have been designed and tested with lysate supplemented with alternative sigma factors, demonstrating the versatility of CFS (Fig. 2A) [44]. These sigma factors have even been used to construct oscillators by controlling each promoter family [40].

Apart from sigma factors, transcription is also controlled by transcription factors or small RNA molecules. Among the numerous transcription factors of *E. coli*, seven global transcription regulators (CRP, FNR, IHF, Fis, ArcA, NarL and Lrp) control the expression of 50 % of all genes in living cells [45]. Many of these, as well as other specific transcription factors have been detected in cell lysate [2]. Rho and NusA proteins were found in high amounts in cell lysate, and were more abundant than NusG. Only small amounts of CRP and FIS were found, while LrP, H-NS, IhF, FnR, ArcA, Cra, and Sox were not detected. RapA and GreA, which release the RNAP, were present, along with trace amounts of LacI and DksA. The presence of DksA in CFS raises the question of the regulation of the native RNAP by the (p)ppGpp alarmone, which may be synthesized by the ribosome-associated RelA protein upon sensing of uncharged transfer RNAs [46]. Detectable amounts of response regulators for three two-component systems were also measured: OmpR, PhoP, and BasR [2]. To our knowledge, this differential loss of transcription factors during lysate preparation is not yet clearly understood. However, it could be explained by the coarse-grained protocols used for lysate preparation, which result into batch-to-batch variability in CFS [47], indicating different protein contents. Concerning small RNA, the degradation of RNA during lysate preparation indicates that small RNA molecules involved in regulating transcription are likely absent in the cell-free preparation, but this has not been conclusively measured as of today.

A remaining question related to mRNA synthesis in CFS concerns the availability of the nucleotides (NTPs), as substrates. All NTPs are provided [4] and their concentrations clearly impact protein production [47], [48]. It is unclear how NTPs abundances change over time in a cell-free reaction. ATP is partially regenerated through pathways like oxydative phosphorylation or glycolysis using 3-PGA or PEP as fuelling molecules which leads to higher yield in protein and mRNA production [5], [14], [49]. Moreover, CFS exhibits an intrinsic capacity to produce ATP, CTP, UTP and GTP from monophosphates (NMPs) when both a phosphate donor and an enery source (glucose or other carbon sources ranging from maltose to glutamate) are provided [41]. Consistently, replacing NTPs with nucleoside monophosphates (NMPs) resulted in equivalent protein synthesis [50]. Nevertheless, most cell-free users currently add NTPs rather than glucose in CFS to produce a sufficient amount of mRNA. Eventually, the decay of mRNA concentration after a few hours suggests that NTPs levels quickly become limiting for sustained mRNA synthesis [50], [51].

The degradation of mRNA can be measured in CFS as in living cells using mRNA-fused aptamers and a dye that links mRNA abundance to a fluorescence signal [35], [52], [53], [54]. Siesgal-Gaskins et al. (2014) used a 35-bases malachite green aptamer (MGapt) sequence that contains a ligand-binding site for the malachite green dye allowing to monitor mRNA abundance over time [35]. The mRNA degradation process can be modelled by a first order reaction as the rate of reaction is proportional to the concentration of the substrate mRNA [35], [36]. The mRNA degradation is reduced in CFS compared to *in vivo*, with a mean half-life of deGFP mRNA around 13 min compared to 6.8 min for total mRNA *in vivo* [55]. Depending on the lysate composition, it was observed that mRNA degradation in CFS is increased when the available pool of ribosomes is insufficient to densely cover the number of transcripts and protect from the endonucleolytic activity [56], [57].

### 1.3 Translation and translational regulation

The translation process can be divided into three steps: initiation, elongation, and termination. Foshag et al. (2018) used media supplemented with glucose to grow *E. coli*, and quantified the proteins necessary for a functional translation process in the lysate [2], [8]. Based on their analysis, components that are essential to translation, such as the ribosome and aminoacyl tRNA synthetases, are significantly abundant. All of the tRNA ligases and synthetases are also abundant, except for the glycine-tRNA ligase subunit GlyQ and the glutamyl-Q tRNA synthetase GluQ. In addition, the initiation factor IF1, IF2, and IF3 abundances are consistent with the optimized ratio used in PURE systems [58], [59]. The elongation factors EF-G, EF-Tu, and EF-Ts are also abundant, but the addition of EF-Tu has been shown to significantly enhance yield. Eventually, the release factors RF2 and RF3, and the ribosome recycling factor, are present at comparable levels in CFS and *in vivo*. However, the release factor RF1, which recognizes the amber stop codon, appears to be less abundant [8]. Changing the composition of the growth medium, strains, or methods used to produce a lysate has an impact on its composition. Contreras-Llano et al. (2020) used consortia of bacterial strains to create cell lysates enriched in 34 essential proteins of the *E. coli* translation machinery. They demonstrated that overexpression of the translation machinery can effectively reprogram the bacterial proteome, leading to changes in the expression levels of over 700 proteins [60].

Omics studies were conducted on distinct lysate preparations, enabling investigation of diverse metabolic pathways maintenance, and highlighting the observed variability based on lysate preparation conditions. Falgenhauer et al. (2021) compared the protein content of a homemade lysate to a commercial one. The two lysates significantly differed in the abundance of nearly half of the detected proteins, in particular proteins of the carbohydrate metabolism, glycolysis, amino acid and nucleotide biosynthesis, the RNA modification and processing, the DNA modification and replication, the transcription initiation and termination as well as the tricarboxylic acid (TCA) cycle [61]. Consistently, Rasor et al. (2023) demonstrated using metabolomic and proteomic analyses that post-lysis processing and buffer composition alter the lysate composition and its activity. When cells are grown in an an amino acid-poor environment like the defined medium EzGlc, an increased prevalence of nearly all amino acid biosynthesis pathways was observed compared to the widely used YTPG medium [62]. This is in agreement with recent modeling that predict de novo amino acid synthesis from glucose through a largely complete TCA cycle, with diversion of metabolic flux into the Entner− Doudoroff pathway to produce NADPH [63]. Optimisation studies have proven there is no need to supplement CFS with tRNA for protein production, suggesting that tRNAs are recycled or that tRNA synthetases are present and active in the lysate [47], [64]. Taken together, the key substrates for translation that are amino acids and tRNA can be regenerated in some CFS.

The most effective extracts for CFS are thought to be those from exponentially growing *E. coli* cells in an effort to capture the most active translation mechanism. Recently, highly active cell-free protein synthesis systems have been obtained from *E. coli* cells harvested under stress and non-growth conditions [39]. The stoichiometry of ribosomes and key translation factors was conserved, and protein synthesis rates were similar to those of lysates obtained from fast-growing cells, together indicating a fully intact translation system. This preparation of non-conventional extracts revealed that the endogenous housekeeping sigma factor, σ^70^, had been replaced in the core RNAP by alternative sigma factors. However, replacement of the σ^70^-specific promoter controlling expression of the gene of interest with a promoter recognised by the stress-induced alternative sigma factor led to protein synthesis comparable to that of conventional lysates. The use of non-conventional extracts increases the range of potential cell-free applications, but also challenges current beliefs about the limitation of the translation apparatus system in cell lysate.

The regulation of bacterial translation is determined by a range of interactions between target mRNAs and various RNA-based regulators, metabolites or RNA-binding proteins [65]. As described in the previous paragraph (§ Transcription and transcriptional regulation), RNA-based regulators and metabolites are likely to be degraded during the lysate preparation. Despite the absence of data concerning RNA-binding proteins in CFS, there is no reason to believe that such proteins are lost.

Beyond the regulation of protein synthesis, protein levels are also set *in vivo* through (i) dilution resulting from cell division and (ii) degradation by proteases. Because regular CFS is a batch reaction, there is no dilution [66]. However, degradation still occurs in CFS, as the *E. coli* lysate contains endogenous proteases, such as DegP, OmpT, or Lon proteases [67] and the ClpXP, ClpAP complexes [56]. The most recent studies on protein degradation that are discussed below have been performed with strains deficient in OmpT and Lon proteases (BL21 DE3, BL21 DE3 STAR, BL21 DE3 Rosetta2). Peptide tags specific to the ClpXP and ClpAP proteases were used to regulate but also to control the degradation rate of eGFP [56]. Karzbrun et al. (2011) measured the degradation of a purified His-eGFP-ssrA protein at various concentrations in CFS and observed a rapid saturation of the degradation rate at 100 nM, with a maximal rate of 12 nM.min^−1^ [1]. ClpXP only degraded up to about 0.5 μM protein, beyond which the rate slowed down considerably or stopped completely. Taken together, proteins are degraded in CFS, although to a lesser extent than *in vivo*.

### 1.4 Protein folding

Cytoplasmic chaperones are generally more abundant in the lysate when compared to *in vivo* conditions. The CFS contains major chaperone systems such as the trigger factor (TF), DnaK/DnaJ, and GroEL/GroES, which play a critical role in the folding of nascent proteins. However, smaller amounts of DnaJ are measured in the lysate. In addition, the CFS contains DsbA and DsbC, which catalyze the formation of disulfide bonds [8].

The concentration of cell lysate does not significantly modify the interaction between a ligand and its target protein [68]. However, increasing the concentration of cell lysate within CFS results in a monotonic increase in the thermodynamic stability of the *Bacillus subtilis* cold shock protein B (*Bs*CspB). The impact of crowding on stability is a result of the high concentration of macromolecules that creates both hard and soft interactions between the crowding agents and protein. Hard interactions lead to volume exclusion, which reduces the space available to the protein and increases its overall thermodynamic stability. Soft interactions involve both stabilizing and destabilizing effects of macromolecules on adjacent proteins [69]. This would suggest that crowding contributes to the overall stabilisation of proteins and protein folding in lysates. Nonetheless, this does not apply to synthesis of membrane proteins, which precipitate in the lysate and require additional *in vitro* folding procedures using lipid vesicles to recover correctly folded proteins [70].

### 1.5 Metabolism and energy production

Energy production in living bacteria takes place through glycolysis, the TCA cycle and the respiratory chain. While the enzymes of the glycolytic and TCA cycle present in the cell cytoplasm are very likely to be conserved during lysate preparation [7], [71], the respiration process could be lost during the multiple centrifugations used to get rid of the cell debris.

Various cell-free recipes using different glycolytic substrates have been developed, indicating that the glycolytic network remains intact [5]. Glucose [51], [63], glucose 6-phosphate [7], fructose-1,6-bisphosphate [72], maltose & maltodextrin [73], creatine phosphate [74], phosphoenolpyruvic acid [51], 3-PGA [75], acetyl phosphate [76], pyruvate [77], glutamate [14], as well as starch and glycogen [78] have been used as energy sources, supporting the idea that any glycolytic intermediate can be used for energy regeneration in CFS. Glycolytic enzymes and their relative abundances have been characterised by tandem mass spectrometry in *E. coli* crude cell extracts [79]. The TCA cycle is considered to be intact in lysate preparations when cells are grown on glucose, because succinate, acetate, malate, oxaloacetate, and aspartate are produced in CFS [14]. It is unclear whether the enzymes of the TCA cycle are abundant enough to be efficient in CFS when the cells are grown in glucose-free conditions. A mutli-omic study measured a higher concentration of succinate when using a glutamate-based washing buffer, supposing a higher TCA cycle activity [80]. Jewett et al. (2008) hypothesized that respiration occurs in inverted membrane vesicles created during cell lysis based on membrane particles still present in the lysate. In a platform designed to mimic more accurately the *E. coli* cytoplasm (the Cytomin system), the authors observed that energy was not only provided by the expected pyruvate metabolism, but also by another pathway. When inhibiting oxydative phosphorylation in several independent ways, they observed a significantly lower protein production and therefore concluded that oxidative phosphorylation is responsible for this additional and unexpected energy production [14]. Although respiration is probably the main pathway for ATP regeneration active in CFS, fermentation could occur too. When preparing the lysate of anaerobically grown *E. coli* cells, one study reports increased production of several reporter proteins probably resulting from fermentative metabolism [79].

### 1.6 Toxicity and resources competition

Heterologous gene expression, while useful in many applications, often generates a significant burden to living cells. This is due to the cost of protein production, which can have a variety of impacts on cell function and growth. In bacteria, this burden is largely attributed to the energy required to produce proteins and the cost of building the full translation machinery, including ribosomes [81], [82], [83]. Additionally, certain key metabolites can be consumed during protein production or when these proteins are active, further straining the cell [84]. Over time, the burden of heterologous gene expression can lead to the production of toxic metabolites [85] or misfolded proteins [86], which can create inclusion bodies and impact growth rates [87].

Several studies have demonstrated that co-expressing various synthetic circuits makes it possible to measure competition for the translation machinery in CFS [16], [35], [63], [88]. As in living cells, during the production of a heterologous protein, the main competition for resources is assumed to be at the translation level.

CFS is not capable of measuring the competition for metabolites that affect cell growth. *In vivo* the depletion of such metabolites leads to a reorganization of the entire cell network that will cause substantial modifications of protein production. In CFS, since growth is no longer relevant, protein production and growth defect are decoupled [16]. This, provides an advantage for bioproduction as most toxic effects are no longer operative in cell-free reactions [16], [89], [90]. However, when using CFS as a prototyping platform, biosensors can be added to the cell-free mix in order to monitor the evolution of specific metabolites and predict their impact on living cells [91]. This approach can be particularly useful for predicting the influence of certain metabolites that may have complex effects on cellular growth and function.

## 2 *In vivo* functions that are mostly defective in lysate-based cell-free systems

### 2.1 Spatial organization

High resolution microscopy of *E. coli* revealed that the nucleoid region, which is rich in DNA, is located at the centre of the cell [92]. Conversely, the majority of ribosomes are spatially separated from the nucleoid region and are located at the cell poles (Fig. 1, “spatial organization” column) [92], [93]. This spatial segregation results in distinct expression profiles for genes located at the periphery of the nucleoid, close to the ribosomes, and for genes located within the nucleoid, further from the ribosomes [94]. *In vivo*, spatial organization relies on the membrane and macromolecular crowding. Crowding plays a major role in the probability of cell compounds binding to each other, as well as in protein folding and stability. Therefore, living cells maintain the overall concentration of macromolecules within a narrow range over time [95]. This control of density is mimicked in CFS by using crowding agents such as PEG [4], [5]. Increasing levels of crowding have been used to facilitate (i) the binding and wrapping of DNA around the inner surface of giant unilamellar vesicles and (ii) the accumulation of DNA close to the surface of small water-in-oil droplets [96]. Chaucan and colleagues (2022) studied the effect of macromolecular crowding on gene expression in vesicles that are of similar size to cells (Fig. 2B). They reported that high crowding impedes the ability of ribosomes to reach mRNA, and that adjusting the level of crowding results in gene expression comparable to that observed *in vivo* with two regions: the outer, ribosome-rich periphery and the inner, ribosome-poor area [97].

Despite the absence of spatial organization in the lysate, the formation of membrane structures through fragmentation and rearrangement of cell membranes has been documented for several decades. This was initially observed in Hertzberg's 1974 study on oxidative phosphorylation using purified cell-free vesicles [98]. These vesicles present in CFS have been characterized using nanocharacterisation techniques, revealing a diameter ranging from 10 to 100 nm and a relatively lower concentration compared to ribosomes and other small complexes. The method of lysate preparation has been shown to play a crucial role, as centrifugation affects vesicle concentration and the lysis method impacts vesicle size. Notably, when the centrifugation speed was reduced, vesicle enrichments of 1.2–2.0 times were observed. Sonicated extracts exhibited an average vesicle size of 130 nm, while homogenized extracts showed an average size of 160 nm, with the presence of multiple discrete vesicle populations [99]. The exact mechanism of vesicle formation during mechanical lysis remains unexplored to the best of our knowledge. However, a recent review about mechanisms of biologically induced lysis reports that Gram-negative bacteria produce membrane vesicles either through detachment of the outer membrane or by explosive cell lysis, and that the biogenesis pathway is of primary importance for understanding the structure and content of vesicles [100]. This suggests that studying vesicle formation in CFS could provide valuable insights, as vesicles are essential for activating cost-effective energy metabolism from oxidative phosphorylation in CFS [99], [100].

### 2.2 Residual homeostasis

Homeostasis refers to the active maintenance of the internal environment through molecular mechanisms, and can be described as a dynamic equilibrium. *In vivo*, bacteria maintain many properties in a statistically stable manner, including growth rate, density, pH, concentrations of the cellular machineries, RNAs, proteins, and metabolites, as well as fluxes [82], [101], [102], [103], [104], [105], [106]. Most of these entities are no longer stabilized in CFS, except for pH that can be artificially maintained to a constant setpoint over time (Fig. 1, “maintaining homeostasis” column). In bacteria, various mechanisms are used to maintain cytoplasmic pH (active H^+^ transport, metabolic reactions, and through passive physiological adaptations) using protein machinery embedded in the cellular membrane. While this membrane-dependent mechanism cannot be used in CFS, a basic solution consists in supplementing the lysate with a buffer. HEPES is a commonly used buffer in CFS with a pKa of 7.5 and useful pH range of 6.8–8.2 [5].

Moreover, although the initial density has been optimized for protein production using crowding agents [4], [5], the density over time has not been monitored in CFS; however, it is anticipated to fluctuate due to changes in metabolite concentration and the production of mRNA and proteins.

Eventually, mRNA, proteins, and ATP can exhibit a short time of constant concentration. The mRNA remains stable for a short while (30 min), during which production compensates for its degradation [35]. Proteins that are not protease-sensitive are expected to maintain a constant level, similar to what has been observed for GFP. ATP concentration remains stable for several minutes [51] to hours [88] due to an equilibrium between regeneration and consumption. Another form of residual homeostasis is allosteric regulation of enzymes that may still provide some passive regulations of metabolism in CFS, but has not been studied in CFS to our knowledge.

### 2.3 Adaptation to changing conditions

Living bacteria are dynamic systems that quickly adapt to changing environments through a range of genetic and molecular mechanisms (Fig. 1, “adaptation to changing conditions” column, first row) [107], [108], [109]. One historical example of bacterial growth adaptation is the diauxic growth, which led to the discovery of gene regulation through the work on the *lac* operon by Burstein et al. in 1965 [110]. To enable adaptation, bacteria require sensors of condition changes that can be made of protein complexes embedded in the membrane like the phosphotransferase sugar uptake system, or of several transcription factors present in the cytoplasm [111]. Once changes are detected, the cell uses global and local regulators organised in large gene-regulatory networks to activate or repress genes [112]. These regulators can eventually target genes in the genome, RNAs, or directly regulate protein conformation and activity [113], [114], [115]. If we consider the notion of ”adaptation” as the ability to sense and adjust the physiology in response to a change in the environment, one may wonder if adaptation still occurs in CFS.

Regarding sensors, CFS does not contain any of the numerous transmembrane sensors that are known to be active in living cells (Fig. 1, “adaptation to changing conditions” column, last row). Even if some membrane structures can still be found in the lysates [98], [99], [100], [116], no native transmembrane sensors leading to gene activation or repression in CFS have yet been identified. This does not prevent CFS to be affected by drastic changes in their environment, like changes in the temperature or the addition of chemicals that can alter the running CFS reaction.

After sensing a change in the environment, *in vivo* adaptation occurs through a switch from an original physiological state to a new one. This requires the presence of regulatory networks, degradation machineries, or dilution mechanisms, three cell processes that are at least partially preserved in CFS. Even if RNA-based regulatory networks are probably disabled because of RNAses within the lysate, protein-based regulatory networks may be operational thanks to the presence of sigma factors, transcription factors, and potentially proteins for activation cascades (§Transcription and transcriptional regulation). In addition, artificial regulatory networks based on RNA or protein cascades have been implemented in cell-free to characterize different regimes [57], [87], [117], [118]. By manipulating regulatory networks, different outputs were observed, but without monitoring a transition over time [119]. Finally, in an attempt to reconstruct a full native regulatory network by supplementing CFS with the entire *E. coli* genome, the profiles of multiple RNAs appeared to be similar to *in vivo* measurements, while protein profiles significantly differed (Fig. 2A) [120]. This suggests a resource limitation in CFS, probably at the translation level, but such an approach may allow for partial reconstitution of a native regulatory network.

On top of regulatory networks, living cells also take advantage of degradation and dilution mechanisms to operate a switch and remove regulators from earlier ages. As CFS still contains DNAses, RNAses and proteases, degradation processes are functional and improvable. Shin and Noireaux (2010) demonstrated that mRNA inactivation rate can be increased using the *E. coli* mRNA interferase MazF, resulting in an accelerated mRNA global turnover. The same is true for protein degradation rate using peptide tags specific to the endogenous *E. coli* AAA+ proteases [56].

In conclusion, CFS are not well-suited to mimic some adaptive behaviors of living cells. This is due to the fact that the lifespan of the batch reaction is limited to 8 h by the depletion of precursors, the decrease in enzymatic activities and the accumulation of byproducts. As a result, the system cannot sustain ongoing reactions beyond this time frame, which is a significant limitation in attempting to simulate the complex and adaptive behaviors exhibited by living cells [4], [35], [121], [122], [123].

### 2.4 Device-implemented homeostasis and adaptation

In order to restore both homeostasis and adaptation observed *in vivo*, much effort has been put into reactors (Fig. 2 C) and engineering microfluidic devices (Fig. 2D) that allow small molecule exchange between CFS and a feed solution. In CFS, adaptation is reduced to the ability of a system to shift from one state to another, without any sensing machinery.

A first possible device for achieving long-term steady-state reactions in a cell-free environment involves the diffusion of small molecules through ultrafiltration membranes (Fig. 2 C, top part) [124], [125], [126]. To do so, a semipermeable compartment is used for encapsulation, allowing users to finely adjust the reaction environment while retaining the transcription-translation properties from CFS [127], [128]. Another approach involves the use of continuous reaction conditions facilitated by protein synthesis in a functionalized phospholipid vesicle, which is surrounded by a feeding solution (Fig. 2 C, bottom part). The reactor obtained thereby can sustain protein expression for up to four days, provided that oxygen diffusion and osmotic pressure are correctly maintained [129]. However, the most advanced approach involves the coupling of microfluidic devices with CFS to maintain longer steady-state reactions and enable monitoring transitions between states (Fig. 2D) [121], [130]. By employing reactors of nanoliters volume size that enable the exchange of small molecules, the synthesis and product removal times can be effectively increased [121], [130], [131], [132]. Using these reactors, steady states were maintained up to 72 hr [121], and regular dilutions were performed in discrete steps, where each dilution step added both a fresh cell-free mix and DNA. They measured the dynamic nature of synthesis and dilution by alternating between periods where DNA template or water was added. This approach allows for the capture of adaptation in cell-free systems through measurements of the circuit's dynamic changes. Multiple transitions have been observed at the transcriptional level (switching from one RNA to another) and translational level (switching from one protein to another) by regulating production, degradation, and dilution [121]. Eventually, it is possible to achieve oscillations using a genetic oscillator that relies on positive feedback and delayed negative feedback. Such construction demonstrates the ability to sustain continuous reaction conditions, allowing for complex dynamics to occur in cell-free systems [121], [130], [131], [133].

## 3 Conclusion and outlook

Lysate-based cell-free systems serve as versatile tools for prototyping genetic circuits in synthetic biology and biotechnology, as well as for decoding fundamental processes of the central dogma and broader aspects of living systems. However, expanding the scope of applications, such as advancing the development of complex synthetic circuits and chassis cells, depends on the ability of cell-free systems to accurately reproduce *in vivo* behaviors. Although the lysate has traditionally been considered an enigmatic black box, mostly due to the uncertainties surrounding the functional properties of living cells lost and preserved in CFS, recent studies have started to clarify this longstanding issue.

The central dogma, which governs DNA expression through transcription and translation, is predominantly preserved in CFS, making them a valuable platform for protein expression. Mathematical models based on CFS data have been successfully tested and demonstrated accurate reproduction of changes in mRNA and protein levels observed in cell-free reactions [134]. Although the process of lysate preparation involves significant physical disruptions that can alter or eliminate certain regulatory mechanisms, protein-based regulations still occur to some extent in CFS. However, RNA-based regulations are believed to be lost in CFS because of the degradation or removal of genome fragments during the lysate preparation. Complex functions found in living cells, such as maintaining metabolic steady state, adapting to environmental changes, and spatial organization, are mostly lost in regular CFS. However, these complex functions can be reimplemented in CFS through innovative semi-continuous devices like microfluidics, ultrafiltration membranes, and encapsulation.

*In vivo*, the redistribution of resources during the implementation of an exogenous circuit results both from the combined effects of the circuit's interaction with the host metabolism and the consequent rewiring of the host metabolism on circuit activity. Currently, only the circuit's interaction with the host metabolism can be evaluated in regular CFS by adding specific biosensors that measure the impact of resource depletion. By reconstructing the transcriptome and proteome in CFS, it may become possible to also evaluate the consequent rewiring of the host metabolism on circuit activity and gain insight into resource allocation. For instance, Deyama et al. (2021) developed the *in vitro* genome transcription-translation (iGeTT) in which they incorporated a complete *E. coli* genome in an encapsulated CFS and demonstrated a correlation between the most expressed genes in CFS and *in vivo* at the mRNA level [120]. By encapsulating CFS within liposomes, it becomes easier to manipulate biological systems and observe their behaviors within the context of cell division. Encapsulation will allow to study membrane proteins, recreate sensor systems coupled to regulatory cascade controlling gene expression, to implement cell-to-cell communication, and to rebuild spatial organization and compartmentalization, among other possibilities.

The ultimate objective is to recreate the entire physiology and behavior of living cells, including regulation and division, *in vitro*. This represents a significant challenge within the field. Accomplishing this objective would enable the development of standardized "synthetic cells" for advanced prototyping purposes. Building upon the foundational work laid by Noireaux's lab [43], [135], various initiatives focused on artificial cell research, such as "Build a cell" in the USA, "SynCellEU" and "fabricell" in Europe, have emerged to tackle this challenge [136], [137], [138]. The initial step involves constructing "synthetic cells" using a bottom-up approach that aims to reactivate the genetic programs responsible for cell division. Ultimately, self-replicating "synthetic cells" will serve as valuable tools for further investigating natural evolution, selection, and adaptation processes.

## Credit authorship contribution statement

**Léa Wagner**: Conceptualization, Investigation, Visualization, Writing – original draft Writing – review & editing. **Matthieu Jules:** Conceptualization, Investigation, Visualization, Supervision, Writing – original draft, Writing – review & editing. **Olivier Borkowski**: Conceptualization, Investigation, Visualization, Supervision, Writing – original draft, Writing – review & editing.

## Declaration of Competing Interest

The authors declare that they have no affiliations with or involvement in any organization or entity with any financial interest in the subject matter or materials discussed in this manuscript.

## References

[1] Karzbrun E., Shin J., Bar-Ziv R.H., Noireaux V. (2011). Coarse-grained dynamics of protein synthesis in a cell-free system. Phys Rev Lett.

[2] Garenne D., Beisel C.L., Noireaux V. (2019). Characterization of the all-*E. coli* transcription-translation system myTXTL by mass spectrometry. Rapid Commun Mass Spectrom.

[3] Garenne D., Haines M.C., Romantseva E.F., Freemont P., Strychalski E.A., Noireaux V. (2021). Cell-free gene expression. Nat Rev Methods Prim.

[4] Sun Z.Z., Hayes C.A., Shin J., Caschera F., Murray R.M., Noireaux V. (2013). Protocols for implementing an *Escherichia coli* based TX-TL cell-free expression system for synthetic biology. J Vis Exp JoVE.

[5] Dopp B.J.L., Tamiev D.D., Reuel N.F. (2019). Cell-free supplement mixtures: elucidating the history and biochemical utility of additives used to support *in vitro* protein synthesis in *E. coli* extract. Biotechnol Adv.

[6] Levine M.Z., Gregorio N.E., Jewett M.C., Watts K.R., Oza J.P. (2019). *Escherichia coli*-based cell-free protein synthesis: protocols for a robust, flexible, and accessible platform technology. JoVE J Vis Exp.

[7] Kim D.-M., Swartz J.R. (2001). Regeneration of adenosine triphosphate from glycolytic intermediates for cell-free protein synthesis. Biotechnol Bioeng.

[8] Foshag D., Henrich E., Hiller E., Schäfer M., Kerger C., Burger-Kentischer A. (2018). The *E. coli* S30 lysate proteome: a prototype for cell-free protein production. New Biotechnol.

[9] Laohakunakorn N., Grasemann L., Lavickova B., Michielin G., Shahein A., Swank Z. (2020). Bottom-up construction of complex biomolecular systems with cell-free synthetic biology. Front Bioeng Biotechnol.

[10] Kohler R.E. (1972). The reception of Eduard Buchner’s discovery of cell-free fermentation. J Hist Biol.

[11] Nirenberg M.W., Matthaei J.H. (1961). The dependence of cell-free protein synthesis in *E. coli* upon naturally occurring or synthetic polyribonucleotides. Proc Natl Acad Sci.

[12] Nirenberg M., Leder P. (1964). RNA codewords and protein synthesis. Science.

[13] Chappell J., Jensen K., Freemont P.S. (2013). Validation of an entirely *in vitro* approach for rapid prototyping of DNA regulatory elements for synthetic biology. Nucleic Acids Res.

[14] Jewett M.C., Calhoun K.A., Voloshin A., Wuu J.J., Swartz J.R. (2008). An integrated cell-free metabolic platform for protein production and synthetic biology. Mol Syst Biol.

[15] Dudley Q.M., Karim A.S., Nash C.J., Jewett M.C. (2020). *In vitro* prototyping of limonene biosynthesis using cell-free protein synthesis. Metab Eng.

[16] Borkowski O., Bricio C., Murgiano M., Rothschild-Mancinelli B., Stan G.-B., Ellis T. (2018). Cell-free prediction of protein expression costs for growing cells. Nat Commun.

[17] Fuciarelli A.F., Sisk E.C., Thomas R.M., Miller D.L. (1995). Induction of base damage in DNA solutions by ultrasonic cavitation. Free Radic Biol Med.

[18] Kim T.-W., Keum J.-W., Oh I.-S., Choi C.-Y., Park C.-G., Kim D.-M. (2006). Simple procedures for the construction of a robust and cost-effective cell-free protein synthesis system. J Biotechnol.

[19] Ahn J.-H., Chu H.-S., Kim T.-W., Oh I.-S., Choi C.-Y., Hahn G.-H. (2005). Cell-free synthesis of recombinant proteins from PCR-amplified genes at a comparable productivity to that of plasmid-based reactions. Biochem Biophys Res Commun.

[20] McSweeney M.A., Styczynski M.P. (2021). Effective use of linear DNA in cell-free expression systems. Front Bioeng Biotechnol.

[21] Batista A.C., Levrier A., Soudier P., Voyvodic P.L., Achmedov T., Reif-Trauttmansdorff T. (2022). Differentially optimized cell-free buffer enables robust expression from unprotected linear DNA in exonuclease-deficient extracts. ACS Synth Biol.

[22] Norouzi M., Panfilov S., Pardee K. (2021). High-efficiency protection of linear DNA in cell-free extracts from *Escherichia coli* and *Vibrio natriegens*. ACS Synth Biol.

[23] Seki E., Matsuda N., Yokoyama S., Kigawa T. (2008). Cell-free protein synthesis system from *Escherichia coli* cells cultured at decreased temperatures improves productivity by decreasing DNA template degradation. Anal Biochem.

[24] Diaz R., Nordström K., Staudenbauer W.L. (1981). Plasmid R1 DNA replication dependent on protein synthesis in cell-free extracts of *E. coli*. Nature.

[25] Diaz R., Staudenbauer W.L. (1982). Replication of the broad host range plasmid RSF 1010 in cell-free extracts of *Escherichia coli* and *Pseudomonas aeruginosa*. Nucleic Acids Res.

[26] del Solar G., Diaz R., Espinosa M. (1987). Replication of the streptococcal plasmid pMV158 and derivatives in cell-free extracts of *Escherichia coli*. Mol Gen Genet MGG.

[27] Staudenbauer W.L., Arber W., Henle W., Hofschneider P.H., Humphrey J.H., Klein J., Koldovský P. (1978). Curr. Top. Microbiol. Immunol..

[28] Rakowski S.A., Filutowicz M. (2013). Plasmid R6K replication control. Plasmid.

[29] Korhonen J.A., Pham X.H., Pellegrini M., Falkenberg M. (2004). Reconstitution of a minimal mtDNA replisome. *Vitr* EMBO.

[30] Fujiwara K., Katayama T., Nomura S.M. (2013). Cooperative working of bacterial chromosome replication proteins generated by a reconstituted protein expression system. Nucleic Acids Res.

[31] Libicher K., Hornberger R., Heymann M., Mutschler H. (2020). *In vitro* self-replication and multicistronic expression of large synthetic genomes. Nat Commun.

[32] Okauchi H., Ichihashi N. (2021). Continuous cell-free replication and evolution of artificial genomic DNA in a compartmentalized gene expression system. ACS Synth Biol.

[33] Silverman A.D., Karim A.S., Jewett M.C. (2020). Cell-free gene expression: an expanded repertoire of applications. Nat Rev Genet.

[34] Shin J., Noireaux V. (2010). Efficient cell-free expression with the endogenous *E. coli* RNA polymerase and sigma factor 70. J Biol Eng.

[35] Siegal-Gaskins D., Tuza Z.A., Kim J., Noireaux V., Murray R.M. (2014). Gene circuit performance characterization and resource usage in a cell-free “breadboard. ACS Synth Biol.

[36] Moore S.J., MacDonald J.T., Wienecke S., Ishwarbhai A., Tsipa A., Aw R. (2018). Rapid acquisition and model-based analysis of cell-free transcription-translation reactions from nonmodel bacteria. Proc Natl Acad Sci USA.

[37] Deich C., Cash B., Sato W., Sharon J., Aufdembrink L., Gaut N.J. (2023). T7Max transcription system. J Biol Eng.

[38] Bervoets I., Charlier D. (2019). Diversity, versatility and complexity of bacterial gene regulation mechanisms: opportunities and drawbacks for applications in synthetic biology. FEMS Microbiol Rev.

[39] Failmezger J., Rauter M., Nitschel R., Kraml M., Siemann-Herzberg M. (2017). Cell-free protein synthesis from non-growing, stressed *Escherichia coli*. Sci Rep.

[40] Yelleswarapu M., van der Linden A.J., van Sluijs B., Pieters P.A., Dubuc E., de Greef T.F.A. (2018). Sigma factor-mediated tuning of bacterial cell-free synthetic genetic oscillators. ACS Synth Biol.

[41] Garamella J., Marshall R., Rustad M., Noireaux V. (2016). The all *E. coli* TX-TL toolbox 2.0: a platform for cell-free synthetic biology. ACS Synth Biol.

[42] Garenne D., Thompson S., Brisson A., Khakimzhan A., Noireaux V. (2021). The all-*E. coli* TXTL toolbox 3.0: new capabilities of a cell-free synthetic biology platform. Synth Biol.

[43] Shin J., Noireaux V. (2012). An *E. coli* cell-free expression toolbox: application to synthetic gene circuits and artificial cells. ACS Synth Biol.

[44] Lin X., Li Z., Li Y., Lu Y. (2021). A robust *Escherichia coli* cell-free expression toolbox driven by sigma factors. Biochem Eng J.

[45] Martínez-Antonio A., Collado-Vides J. (2003). Identifying global regulators in transcriptional regulatory networks in bacteria. Curr Opin Microbiol.

[46] Wendrich T.M., Blaha G., Wilson D.N., Marahiel M.A., Nierhaus K.H. (2002). Dissection of the mechanism for the stringent actor RelA. Mol Cell.

[47] Borkowski O., Koch M., Zettor A., Pandi A., Batista A.C., Soudier P. (2020). Large scale active-learning-guided exploration for *in vitro* protein production optimization. Nat Commun.

[48] Takahashi K., Sato G., Doi N., Fujiwara K. (2021). A relationship between NTP and cell extract concentration for cell-free protein expression. Life.

[49] Alissandratos A., Caron K., Loan T.D., Hennessy J.E., Easton C.J. (2016). ATP recycling with cell lysate for enzyme-catalyzed chemical synthesis, protein expression and PCR. ACS Chem Biol.

[50] Calhoun K.A., Swartz J.R. (2005). Energizing cell-free protein synthesis with glucose metabolism. Biotechnol Bioeng.

[51] Jewett M.C., Swartz J.R. (2004). Substrate replenishment extends protein synthesis with an *in vitro* translation system designed to mimic the cytoplasm. Biotechnol Bioeng.

[52] Paige J.S., Wu K.Y., Jaffrey S.R. (2011). RNA mimics of green fluorescent protein. Science.

[53] Pothoulakis G., Ceroni F., Reeve B., Ellis T. (2014). The spinach RNA aptamer as a characterization tool for synthetic biology. ACS Synth Biol.

[54] Wick S., Walsh D.I.I., Bobrow J., Hamad-Schifferli K., Kong D.S., Thorsen T. (2019). PERSIA for direct fluorescence measurements of transcription, translation, and enzyme activity in cell-free systems. ACS Synth Biol.

[55] Selinger D.W., Saxena R.M., Cheung K.J., Church G.M., Rosenow C. (2003). Global RNA half-life analysis in *Escherichia coli* reveals positional patterns of transcript degradation. Genome Res.

[56] Shin J., Noireaux V. (2010). Study of messenger RNA inactivation and protein degradation in an *Escherichia coli* cell-free expression system. J Biol Eng.

[57] Karig D.K., Iyer S., Simpson M.L., Doktycz M.J. (2012). Expression optimization and synthetic gene networks in cell-free systems. Nucleic Acids Res.

[58] Shimizu Y., Inoue A., Tomari Y., Suzuki T., Yokogawa T., Nishikawa K. (2001). Cell-free translation reconstituted with purified components. Nat Biotechnol.

[59] Li J., Gu L., Aach J., Church G.M. (2014). Improved cell-free RNA and protein synthesis system. PLOS ONE.

[60] Contreras-Llano L.E., Meyer C., Liu Y., Sarker M., Lim S., Longo M.L. (2020). Holistic engineering of cell-free systems through proteome-reprogramming synthetic circuits. Nat Commun.

[61] Falgenhauer E., von Schönberg S., Meng C., Mückl A., Vogele K., Emslander Q. (2021). Evaluation of an *E. coli* cell extract prepared by lysozyme-assisted sonication via gene expression, phage assembly and proteomics. ChemBioChem.

[62] Mohr B., Giannone R.J., Hettich R.L., Doktycz M.J. (2020). Targeted growth medium dropouts promote aromatic compound synthesis in crude *E. coli* cell-free systems. ACS Synth Biol.

[63] Vilkhovoy M., Horvath N., Shih C.-H., Wayman J.A., Calhoun K., Swartz J. (2018). Sequence specific modeling of *E. coli* cell-free protein synthesis. ACS Synth Biol.

[64] Cai Q., Hanson J.A., Steiner A.R., Tran C., Masikat M.R., Chen R. (2015). A simplified and robust protocol for immunoglobulin expression in *Escherichia coli* cell-free protein synthesis systems. Biotechnol Prog.

[65] Jeong Y., Shin H., Seo S.W., Kim D., Cho S., Cho B.-K. (2017). Elucidation of bacterial translation regulatory networks. Curr Opin Syst Biol.

[66] Carlson E.D., Gan R., Hodgman C.E., Jewett M.C. (2012). Cell-free protein synthesis: applications come of age. Biotechnol Adv.

[67] Jiang X., Oohira K., Iwasaki Y., Nakano H., Ichihara S., Yamane T. (2002). Reduction of protein degradation by use of protease-deficient mutants in cell-free protein synthesis system of *Escherichia coli*. J Biosci Bioeng.

[68] Welte H., Sinn P., Kovermann M. (2021). Fluorine NMR spectroscopy enables to quantify the affinity between DNA and proteins in cell lysate. ChemBioChem.

[69] Welte H., Kovermann M. (2020). Insights into protein stability in cell lysate by 19F NMR spectroscopy. Chembiochem.

[70] Focke P.J., Hein C., Hoffmann B., Matulef K., Bernhard F., Dötsch V. (2016). Combining *in vitro* folding with cell free protein synthesis for membrane protein expression. Biochemistry.

[71] Kim D.-M., Swartz J.R. (2000). Prolonging cell-free protein synthesis by selective reagent additions. Biotechnol Prog.

[72] Kim T.-W., Keum J.-W., Oh I.-S., Choi C.-Y., Kim H.-C., Kim D.-M. (2007). An economical and highly productive cell-free protein synthesis system utilizing fructose-1,6-bisphosphate as an energy source. J Biotechnol.

[73] Caschera F., Noireaux V. (2014). Synthesis of 2.3 mg/ml of protein with an all *Escherichia coli* cell-free transcription–translation system. Biochimie.

[74] Kim T.-W., Oh I.-S., Keum J.-W., Kwon Y.-C., Byun J.-Y., Lee K.-H. (2007). Prolonged cell-free protein synthesis using dual energy sources: combined use of creatine phosphate and glucose for the efficient supply of ATP and retarded accumulation of phosphate. Biotechnol Bioeng.

[75] Sitaraman K., Esposito D., Klarmann G., Le Grice S.F., Hartley J.L., Chatterjee D.K. (2004). A novel cell-free protein synthesis system. J Biotechnol.

[76] Ryabova L.A., Vinokurov L.M., Shekhovtsova E.A., Alakhov Y.B., Spirin A.S. (1995). Acetyl phosphate as an energy source for bacterial cell-free translation systems. Anal Biochem.

[77] Kim D.-M., Swartz J.R. (1999). Prolonging cell-free protein synthesis with a novel ATP regeneration system. Biotechnol Bioeng.

[78] Kim H.-C., Kim T.-W., Kim D.-M. (2011). Prolonged production of proteins in a cell-free protein synthesis system using polymeric carbohydrates as an energy source. Process Biochem.

[79] Garcia D.C., Mohr B.P., Dovgan J.T., Hurst G.B., Standaert R.F., Doktycz M.J. (2018). Elucidating the potential of crude cell extracts for producing pyruvate from glucose. Synth Biol.

[80] Rasor B.J., Chirania P., Rybnicky G.A., Giannone R.J., Engle N.L., Tschaplinski T.J. (2023). Mechanistic insights into cell-free gene expression through an integrated -omics analysis of extract processing methods. ACS Synth Biol.

[81] Ceroni F., Algar R., Stan G.-B., Ellis T. (2015). Quantifying cellular capacity identifies gene expression designs with reduced burden. Nat Methods.

[82] Borkowski O., Ceroni F., Stan G.-B., Ellis T. (2016). Overloaded and stressed: whole-cell considerations for bacterial synthetic biology. Curr Opin Microbiol.

[83] Boo A., Ellis T., Stan G.-B. (2019). Host-aware synthetic biology. Curr Opin Syst Biol.

[84] Miguez A.M., McNerney M.P., Styczynski M.P. (2019). Metabolic profiling of *Escherichia coli*-based cell-free expression systems for process optimization. Ind Eng Chem Res.

[85] Martin V.J.J., Pitera D.J., Withers S.T., Newman J.D., Keasling J.D. (2003). Engineering a mevalonate pathway in *Escherichia coli* for production of terpenoids. Nat Biotechnol.

[86] Singh S.M., Panda A.K. (2005). Solubilization and refolding of bacterial inclusion body proteins. J Biosci Bioeng.

[87] Noireaux V., Bar-Ziv R., Libchaber A. (2003). Principles of cell-free genetic circuit assembly. Proc Natl Acad Sci.

[88] Horvath N., Vilkhovoy M., Wayman J.A., Calhoun K., Swartz J., Varner J.D. (2020). Toward a genome scale sequence specific dynamic model of cell-free protein synthesis in *Escherichia coli*. Metab Eng Commun.

[89] Worst E.G., Exner M.P., De Simone A., Schenkelberger M., Noireaux V., Budisa N. (2015). Cell-free expression with the toxic amino acid canavanine. Bioorg Med Chem Lett.

[90] Casini A., Chang F.-Y., Eluere R., King A.M., Young E.M., Dudley Q.M. (2018). A pressure test to make 10 molecules in 90 days: external evaluation of methods to engineer biology. J Am Chem Soc.

[91] Voyvodic P.L., Pandi A., Koch M., Conejero I., Valjent E., Courtet P. (2019). Plug-and-play metabolic transducers expand the chemical detection space of cell-free biosensors. Nat Commun.

[92] Bakshi S., Siryaporn A., Goulian M., Weisshaar J.C. (2012). Superresolution imaging of ribosomes and RNA polymerase in live *Escherichia coli* cells. Mol Microbiol.

[93] Castellana M., Hsin-Jung Li S., Wingreen N.S. (2016). Spatial organization of bacterial transcription and translation. Proc Natl Acad Sci.

[94] Montero Llopis P., Jackson A.F., Sliusarenko O., Surovtsev I., Heinritz J., Emonet T. (2010). Spatial organization of the flow of genetic information in bacteria. Nature.

[95] van den Berg J., Boersma A.J., Poolman B. (2017). Microorganisms maintain crowding homeostasis. Nat Rev Microbiol.

[96] Tsugane M., Suzuki H. (2020). Elucidating the membrane dynamics and encapsulation mechanism of large DNA molecules under molecular crowding conditions using giant unilamellar vesicles. ACS Synth Biol.

[97] Chauhan G., Norred S.E., Dabbs R.M., Caveney P.M., George J.K.V., Collier C.P. (2022). Crowding-induced spatial organization of gene expression in cell-sized vesicles. ACS Synth Biol.

[98] Hertzberg E.L., Hinkle P.C. (1974). Oxidative phosphorylation and proton translocation in membrane vesicles prepared from *Escherichia coli*. Biochem Biophys Res Commun.

[99] Hershewe J.M., Warfel K.F., Iyer S.M., Peruzzi J.A., Sullivan C.J., Roth E.W. (2021). Improving cell-free glycoprotein synthesis by characterizing and enriching native membrane vesicles. Nat Commun.

[100] Toyofuku M., Schild S., Kaparakis-Liaskos M., Eberl L. (2023). Composition and functions of bacterial membrane vesicles. Nat Rev Microbiol.

[101] Schaechter M., MaalØe O., Kjeldgaard N.O. (1958). Dependency on medium and temperature of cell size and chemical composition during balanced growth of *Salmonella typhimurium*. Microbiology.

[102] Cooper S., Helmstetter C.E. (1968). Chromosome replication and the division cycle of *Escherichia coli* B/r. J Mol Biol.

[103] Scott M., Hwa T. (2011). Bacterial growth laws and their applications. Curr Opin Biotechnol.

[104] Goelzer A., Muntel J., Chubukov V., Jules M., Prestel E., Nölker R. (2015). Quantitative prediction of genome-wide resource allocation in bacteria. Metab Eng.

[105] Hui S., Silverman J.M., Chen S.S., Erickson D.W., Basan M., Wang J. (2015). Quantitative proteomic analysis reveals a simple strategy of global resource allocation in bacteria. Mol Syst Biol.

[106] Deloupy A., Sauveplane V., Robert J., Aymerich S., Jules M., Robert L. (2020). Extrinsic noise prevents the independent tuning of gene expression noise and protein mean abundance in bacteria. Sci Adv.

[107] Ishii N., Nakahigashi K., Baba T., Robert M., Soga T., Kanai A. (2007). Multiple high-throughput analyses monitor the response of *E. coli* to perturbations. Science.

[108] Buescher J.M., Liebermeister W., Jules M., Uhr M., Muntel J., Botella E. (2012). Global network reorganization during dynamic adaptations of *Bacillus subtilis* metabolism. Science.

[109] Planson A.-G., Sauveplane V., Dervyn E., Jules M. (2020). Bacterial growth physiology and RNA metabolism. Biochim Biophys Acta BBA - Gene Regul Mech.

[110] Burstein C., Cohn M., Kepes A., Monod J. (1965). Rôle du lactose et de ses produits métaboliques dans l′induction de l′opéron lactose chez *Escherichia coli*. Biochim Biophys Acta BBA - Nucleic Acids Protein Synth.

[111] Kotte O., Zaugg J.B., Heinemann M. (2010). Bacterial adaptation through distributed sensing of metabolic fluxes. Mol Syst Biol.

[112] Salgado H., Santos-Zavaleta A., Gama-Castro S., Peralta-Gil M., Peñaloza-Spínola M.I., Martínez-Antonio A. (2006). The comprehensive updated regulatory network of *Escherichia coli* K-12. BMC Bioinforma.

[113] Stock A.M., Robinson V.L., Goudreau P.N. (2000). Two-component signal transduction. Annu Rev Biochem.

[114] Cases I., de Lorenzo V. (2005). Promoters in the environment: transcriptional regulation in its natural context. Nat Rev Microbiol.

[115] Waters L.S., Storz G. (2009). Regulatory RNAs in bacteria. Cell.

[116] Goerke A.R., Swartz J.R. (2008). Development of cell-free protein synthesis platforms for disulfide bonded proteins. Biotechnol Bioeng.

[117] Takahashi M.K., Hayes C.A., Chappell J., Sun Z.Z., Murray R.M., Noireaux V. (2015). Characterizing and prototyping genetic networks with cell-free transcription–translation reactions. Methods.

[118] Guo S., Murray R.M. (2019). Construction of incoherent feedforward loop circuits in a cell-free system and in cells. ACS Synth Biol.

[119] Singhal V., Tuza Z.A., Sun Z.Z., Murray R.M. (2021). A MATLAB toolbox for modeling genetic circuits in cell-free systems. Synth Biol Oxf Engl.

[120] Deyama T., Matsui Y., Chadani Y., Sekine Y., Doi N., Fujiwara K. (2021). Transcription–translation of the Escherichia coli genome within artificial cells. Chem Commun.

[121] Niederholtmeyer H., Stepanova V., Maerkl S.J. (2013). Implementation of cell-free biological networks at steady state. Proc Natl Acad Sci.

[122] Pandi A., Grigoras I., Borkowski O., Faulon J.-L. (2019). Optimizing cell-free biosensors to monitor enzymatic production. ACS Synth Biol.

[123] Vezeau G.E., Salis H.M. (2021). Tuning cell-free composition controls the time delay, dynamics, and productivity of TX-TL expression. ACS Synth Biol.

[124] Spirin A.S., Baranov V.I., Ryabova L.A., Ovodov S., Alakhov Y.B. (1988). A continuous cell-free translation system capable of producing polypeptides in high yield. Science.

[125] Spirin A.S. (2004). High-throughput cell-free systems for synthesis of functionally active proteins. Trends Biotechnol.

[126] Schwarz D., Junge F., Durst F., Frölich N., Schneider B., Reckel S. (2007). Preparative scale expression of membrane proteins in *Escherichia coli*-based continuous exchange cell-free systems. Nat Protoc.

[127] Boyd M.A., Kamat N.P. (2021). Designing artificial cells towards a new generation of biosensors. Trends Biotechnol.

[128] Boyd M.A., Thavarajah W., Lucks J.B., Kamat N.P. (2023). Robust and tunable performance of a cell-free biosensor encapsulated in lipid vesicles. Sci Adv.

[129] Noireaux V., Libchaber A. (2004). A vesicle bioreactor as a step toward an artificial cell assembly. Proc Natl Acad Sci.

[130] Niederholtmeyer H., Sun Z.Z., Hori Y., Yeung E., Verpoorte A., Murray R.M. (2015). Rapid cell-free forward engineering of novel genetic ring oscillators. ELife.

[131] Karzbrun E., Tayar A.M., Noireaux V., Bar-Ziv R.H. (2014). Programmable on-chip DNA compartments as artificial cells. Science.

[132] van Sluijs B., Maas R.J.M., van der Linden A.J., de Greef T.F.A., Huck W.T.S. (2022). A microfluidic optimal experimental design platform for forward design of cell-free genetic networks. Nat Commun.

[133] Agrawal D.K., Marshall R., Noireaux V., Sontag E.D. (2019). *In vitro* implementation of robust gene regulation in a synthetic biomolecular integral controller. Nat Commun.

[134] Koch M., Faulon J.-L., Borkowski O. (2018). Models for cell-free synthetic biology: make prototyping easier, better, and faster. Front Bioeng Biotechnol.

[135] Garamella J., Garenne D., Noireaux V. (2019). TXTL-based approach to synthetic cells. Methods Enzym.

[136] Gaut N.J., Gomez-Garcia J., Heili J.M., Cash B., Han Q., Engelhart A.E., Adamala K.P. (2022). Programmable fusion and differentiation of synthetic minimal cells. ACS Synth Biol.

[137] Ganar K.A., Leijten L., Deshpande S. (2022). Actinosomes: condensate-templated containers for engineering synthetic cells. ACS Synth Biol.

[138] Allen M.E., James W.H., Divesh K.B., Oscar C., Yuval E. (2022). Hydrogels as functional components in artificial cell systems. Nat Rev Chem.

